# Clopidogrel-Induced Thrombotic Thrombocytopenic Purpura–Hemolytic Uremic Syndrome After Coronary Artery Stenting

**DOI:** 10.1592/phco.24.6.664.34732

**Published:** 2012-01-16

**Authors:** Shawn M Manor, Gregory S Guillory, Suresh P Jain

**Affiliations:** 1 School of Pharmacy, University of LouisianaMonroe, Louisiana; 2 Medical Center of LouisianaNew Orleans, Louisiana; 3 Department of Cardiology, Louisiana State University Health Sciences Center of New OrleansNew Orleans, Louisiana

**Keywords:** clopidogrel, thrombotic thrombocytopenic purpura, hemolytic uremic syndrome

## Abstract

The antiplatelet drug clopidogrel has largely replaced ticlopidine, due to an association between ticlopidine and thrombotic thrombocytopenic purpura–hemolytic uremic syndrome (TTP-HUS). Clopidogrel at first was thought to be void of this potentially fatal adverse effect, but recent case reports have called that assumption into question. Even with proper treatment (plasma exchange), TTP-HUS can persist for weeks. Clinicians should be aware of this possible adverse effect because prompt therapy is imperative for patients' survival. Earlier reports of clopidogrel-related TTP-HUS have involved patients who had received at least 72 hours of therapy. We describe a case of TTP-HUS in a patient who had received only a 300-mg loading dose of clopidogrel.

Clopidogrel has largely replaced ticlopidine as the antiplatelet agent of choice for patients undergoing coronary artery stenting. Clopidogrel also is used to reduce atherosclerotic events in patients with established peripheral arterial disease or after myocardial infarction or recent stroke. Both clopidogrel and ticlopidine inhibit binding of adenosine 5′-diphosphate to its platelet receptor.^1^ Clopidogrel has an improved safety profile compared with that of ticlopidine; however, recent reports have implicated clopidogrel as a possible causative agent of thrombotic thrombocytopenic purpura-hemolytic uremic syndrome (TTP-HUS), which is the most severe adverse reaction associated with ticlopidine.^1^–^4^

## Case Report

A 55-year-old African-American man was From the School of Pharmacy, University of Louisiana, Monroe, Louisiana (Drs. Manor and Guillory); the Medical Center of Louisiana, New Orleans, Louisiana (Dr. Guillory); and the Department of Cardiology, Louisiana State University Health Sciences Center of New Orleans, New Orleans, Louisiana (Dr. Jain). Address reprint requests to Suresh Jain, M.D., Louisiana State University Health Sciences Center, 2025 Gravier Street, Suite 606, New Orleans, LA 70112-2822. admitted to the hospital for cardiac catheterization. His medical history included hypertension, hypercholesterolemia, left ventricular dysfunction, hepatitis C, and a coronary bypass graft 8 months earlier. He also had a history of smoking cigars and marijuana. His drug therapy consisted of aspirin 325 mg day, amlodipine 10 mg day, simvastatin 40 mg at bedtime, isosorbide mononitrate 30 mg day, lisinopril 40 mg day, and zolpidem 5 mg at bedtime as needed. His preadmission laboratory tests showed a white blood cell count of 6.0 x 10^3^ mm^3^ (normal 4.5-11 x 10^3^ mm^3^), hemoglobin level 13.5 mg dl (12-16 mg dl), hematocrit 41% (35-46%), platelet count 195 x 10^3^ mm^3^ (130-400 x 10^3^ mm^3^), blood urea nitrogen (BUN) level 12 mg dl (7-25 mg dl), and serum creatinine level 1.3 mg dl (0.7-1.2 mg dl) (Table 1).

**Table 1:** Chronicle of Laboratory Values

Hospital Day	Time	Hemoglobin (mg/dl)	Hematocrit (%)	BUN (mg/dl)	Creatinine (mg/dl)	Platelet Count (x 10^3^/mm^3^)
1	Preadmission	13.5	41	12	1.3	195
2	8:00 A.M	13.1	40	17	1.4	18
3	6:30 A.M	13.4	39.4	36	3.1	31
3	3:00 P.M	10.7	31.6	9	3.3	34
5	8:00 A.M	7.4	21.5	44	3.0	45
6	8:00 A.M	7.7	22.2	40	2.5	68
14	8:00 A.M	8.9	26.5	52	1.8	239

An intravascular ultrasound was performed, and a stent was placed in the left anterior descending artery. Heparin 7000 U was administered during and after the procedure. A bolus of eptifibatide 180 μg kg was infused, followed by a 2- μg kg minute eptifibatide infusion. The patient also received a loading dose of clopidogrel 300 mg, and clopidogrel 75 mg day was prescribed, along with his previous drug therapy On the morning of hospital day 2, the patient began vomiting (4 times in 12 hrs) and became diaphoretic and short of breath. The only appreciable change in his laboratory values was a decreased platelet count (from 195 x 10^3^ mm^3^ before admission to 18 x 10^3^ mm^3^). At that time it was believed that he was experiencing heparin- induced thrombocytopenia (HIT); therefore, eptifibatide, aspirin, and clopidogrel were discontinued. Testing for HIT antibodies was performed, and the eventual result was negative. Ten units of platelets were infused, and clonidine was started due to an increase in the patient's blood pressure to 172 100 mm Hg. The patient was transferred to the medical intensive care unit.

On hospital day 3, the patient's condition continued to deteriorate. Laboratory values indicated an increase in white blood cell count to 16.3 x 10^3^ mm^3^ (from 11.1 x 110^3^ mm^3^), BUN level rose to 36 mg dl (from 17 mg dl), and creatinine level increased to 3.1 mg dl (from 1.4 mg dl). In addition, the patient's lactate dehydrogenase (LDH) was 1359 U L (normal < 201 U L), and schistocytes were reported on the differential, suggesting hemolysis. The platelet count increased to 31 x 10^3^ mm^3^, but this was attributed to the infusion of platelets the previous evening. Later on day 3, the patient's hemoglobin level decreased to 10.7 mg dl (from 13.4 mg dl), and his hematocrit fell to 31.6% (from 39.4%). A urinalysis was positive for protein, blood, and casts; these findings were further indications of acute renal failure. Due to the patient's decreased renal function, lisinopril was discontinued. A haptoglobin level was obtained, and the reported value of 9 mg dl (normal 30-45 mg dl) was indicative of intravascular hemolysis.^5^ Plasma exchange therapy was started that afternoon (1 L of 5% albumin and 4 L of fresh frozen plasma day), as the working diagnosis was changed to TTP-HUS. Intravenous methylprednisolone 60 mg every 8 hours also was started.

Hemoglobin and hematocrit values reached their lowest on day 5, at 7.4 mg dl and 21.5%, respectively. This prompted the transfusion of 2 units of packed red blood cells. On day 6, blood pressure had stabilized at 143 70 mm Hg, and the patient's platelet count maintained an upward trend (68 x 10^3^ mm^3^). The patient was transferred back to the ward that afternoon, and he complained of continued insomnia. Zolpidem was increased to 10 mg as needed, and later that night the patient experienced auditory hallucinations. Case reports have shown a possible link between zolpidem and hallucinations at doses recommended by the manufacturer.^6^ However, most reports of psychotic reactions to zolpidem involve visual rather than auditory hallucinations. Because the hallucinations ended when zolpidem was discontinued, it is assumed that this central nervous system manifestation was not due to TTP-HUS.^7^

Although most of the patient's laboratory values were trending toward normal (LDH, haptoglobin, hemoglobin, hematocrit), his white blood cell count continued to rise (26.3 x 10^3^ mm^3^ on day 9), without fever and with multiple negative blood cultures. This leukocytosis was attributed to the methylprednisolone. Plasma exchange therapy was discontinued on day 10, and 2 more units of packed red blood cells were transfused. On day 14, the patient's platelet count was 239 x 10^3^ mm^3^, hemoglobin 8.9 mg dl, hematocrit 26.5%, BUN level 52 mg dl, serum creatinine level 1.8 mg dl, and LDH level 154 U L. The patient was discharged on day 14 with the following drug regimen: amlodipine 10 mg day, isosorbide mononitrate 60 mg day, simvastatin 40 mg nightly, aspirin 81 mg day, famotidine 20 mg twice day, and dexamethasone 6 mg day for 1 week.

## Discussion

Both TTP and HUS are multisystem disorders characterized by microangiopathic hemolytic anemia, thrombocytopenia, fever, neurologic manifestations, and renal insufficiency. All of these symptoms result from platelet agglutination. Possible causes of TTP include pregnancy, bacterial endocarditis, autoimmune diseases, neoplasms, bone marrow transplants, and certain drugs (e.g., sulfonamides, iodine, oral contraceptives, antineoplastic agents, ticlopidine). The thrombi associated with TTP-HUS have been shown to contain mostly von Willebrand factor (vWF) and platelets. Evidence points to deficient activity of the vWF cleaving process as a possible cause. However, a recent study suggested that this deficiency in vWF protease activity, although present in TTP, is not necessarily associated with HUS.^8^

Diagnosis of TTP-HUS is based on nonspecific laboratory and clinical findings. Possible signs and symptoms are malaise, headache, fever, confusion, oliguria, visual disturbances, fatigue, abdominal pain, nausea, and vomiting. Laboratory abnormalities include anemia, fragmented red blood cells (schistocytes), elevated reticulocyte count, elevated indirect bilirubin, and elevated LDH level. Coagulation studies are typically normal, especially in the early stages of the disorder.^2^,^5^ In TTP, neurologic dysfunction is typically a predominant symptom, whereas HUS primarily manifests with acute renal failure resulting from renal cell injury. The many similarities between the two disorders make it difficult to distinguish between them.^2^,^3^,^5^ ^9^ Because our patient had major renal manifestations and no neurologic dysfunction, it is unclear whether the final diagnosis should be TTP or HUS. Differentiation was further clouded because testing for vWF protease activity was not performed.

Clopidogrel is considered clinically preferable to ticlopidine due to its favorable safety profile.^2^ About 1 in 5000 patients treated with ticlopidine develop TTP^10^ Phase III trials involving 20,000 patients treated with clopidogrel yielded no reports of TTP-HUS. However, now that clopidogrel has been widely used, case reports have implicated it as a cause of TTP-HUS.^1^-^4^, ^8^ ^11^ One group of researchers reported 11 patients who developed TTP-HUS after receiving clopidogrel. Ten of these patients developed TTP-HUS within 2 weeks after the start of clopidogrel therapy, and in no instance did any patient develop TTP-HUS with less than 3 days of therapy. In this study, three patients received clopidogrel after coronary stenting, and five of the affected patients had been taking lipid- lowering agents, such as atorvastatin or simvastatin.^3^ A case report of simvastatin- induced TTP has been published; however, because our patient continued taking simvastatin, it was ruled out as a causative agent of his TTP- HUS.^12^ Given the time relationship between eptifibatide administration and the precipitous drop in the patient's platelet count, it is possible that the thrombocytopenia may have been induced or exacerbated by eptifibatide. Eptifibatide has been implicated as a causative agent in thrombocytopenia, but it has not been linked to TTP-HUS. Moreover, thrombocytopenia associated with eptifibatide usually resolves spontaneously after the drug is discontinued.^13^

If left untreated, TTP-HUS has a high mortality rate. Plasma exchange therapy has a high response rate and improves short-term survival. Therefore, this therapy should be started as soon as the diagnosis is made.^14^ Plasma exchange is thought to replenish levels of vWF cleaving protease.^5^ The treatment should be administered daily and continued until the platelet count and LDH level normalize, signifying a decrease in ischemia and hemolysis.^5^, ^15^ Response rates vary from patient to patient. Generally, patients achieve clinical remission in the first 1-2 weeks, but some patients may require several weeks of therapy.^16^ Recent studies involving the addition of steroids to plasma exchange have demonstrated no differences in response rates.^17^

## Conclusion

This case report suggests that clopidogrel may be associated with TTP-HUS. With the increasing number of coronary stent procedures performed annually, the potential exists for a higher prevalence of clopidogrel-associated TTP- HUS. Although there are only a few documented cases of clopidogrel-induced TTP-HUS, growing awareness of the possible problem may result in increased reporting.
